# Genetic diversity and association mapping of mineral element concentrations in spinach leaves

**DOI:** 10.1186/s12864-017-4297-y

**Published:** 2017-12-04

**Authors:** Jun Qin, Ainong Shi, Beiquan Mou, Michael A. Grusak, Yuejin Weng, Waltram Ravelombola, Gehendra Bhattarai, Lingdi Dong, Wei Yang

**Affiliations:** 1 Department of Horticulture, University of Arkansas, Fayetteville, AR 72701 USA; 2 Crop Improvement and Protection Research Unit, USDA-ARS, Salinas, CA 93905 USA; 3 USDA-ARS Red River Valley Agricultural Research Center, Fargo, ND 58102 USA

**Keywords:** Genome-wide association study (GWAS), Genotyping by sequencing (GBS), Mineral elements, Single nucleotide polymorphism (SNP), *Spinacia oleracea* L., Spinach

## Abstract

**Background:**

Spinach is a useful source of dietary vitamins and mineral elements. Breeding new spinach cultivars with high nutritional value is one of the main goals in spinach breeding programs worldwide, and identification of single nucleotide polymorphism (SNP) markers for mineral element concentrations is necessary to support spinach molecular breeding. The purpose of this study was to conduct a genome-wide association study (GWAS) and to identify SNP markers associated with mineral elements in the USDA-GRIN spinach germplasm collection.

**Results:**

A total of 14 mineral elements: boron (B), calcium (Ca), cobalt (Co), copper (Cu), iron (Fe), potassium (K), magnesium (Mg), manganese (Mn), molybdenum (Mo), sodium (Na), nickel (Ni), phosphorus (P), sulfur (S), and zinc (Zn) were evaluated in 292 spinach accessions originally collected from 29 countries. Significant genetic variations were found among the tested genotypes as evidenced by the 2 to 42 times difference in mineral concentrations. A total of 2402 SNPs identified from genotyping by sequencing (GBS) approach were used for genetic diversity and GWAS. Six statistical methods were used for association analysis. Forty-five SNP markers were identified to be strongly associated with the concentrations of 13 mineral elements. Only two weakly associated SNP markers were associated with K concentration. Co-localized SNPs for different elemental concentrations were discovered in this research. Three SNP markers, AYZV02017731_40, AYZV02094133_57, and AYZV02281036_185 were identified to be associated with concentrations of four mineral components, Co, Mn, S, and Zn. There is a high validating correlation coefficient with *r* > 0.7 among concentrations of the four elements. Thirty-one spinach accessions, which rank in the top three highest concentrations in each of the 14 mineral elements, were identified as potential parents for spinach breeding programs in the future.

**Conclusions:**

The 45 SNP markers strongly associated with the concentrations of the 13 mineral elements: B, Ca, Co, Cu, Fe, Mg, Mn, Mo, Na, Ni, P, S, and Zn could be used in breeding programs to improve the nutritional quality of spinach through marker-assisted selection (MAS). The 31 spinach accessions with high concentrations of one to several mineral elements can be used as potential parents for spinach breeding programs*.*

**Electronic supplementary material:**

The online version of this article (10.1186/s12864-017-4297-y) contains supplementary material, which is available to authorized users.

## Background

Spinach (*Spinacia oleracea* L., 2n = 2× = 12) is an economically important vegetable crop worldwide with an estimated annual value of $11.8 billion. The United States (US) is the second largest producer of spinach after China with over 550,000 tons harvested, valued at over $300 million annually since 2009 [1, 2]. In addition to its economic importance, spinach is one of the rising vegetable crops in the US in terms of per capita consumption and is considered a healthy vegetable for humans as it is a source of vitamins and mineral nutrients, as well as several health-promoting phytochemicals [3, 4].

Minerals originate in the earth and cannot be made by living organisms [5]. Mineral elements are present in different forms in nature and some of these elements are essential for the body to perform different functions [6]. Most of them mediate vital biochemical reactions by acting as a cofactor or catalyst for many enzymes. They also act as centers of building stabilizing structures such as enzymes and proteins. The five major minerals in the human body are calcium (Ca), phosphorus (P), potassium (K), sodium (Na), and magnesium (Mg) [6, 7]. All of the remaining elements in a human body are called “trace elements”. The trace elements that have a specific biochemical function in the human body are iron (Fe), cobalt (Co), copper (Cu), zinc (Zn), manganese (Mg), molybdenum (Mo), iodine (I), and selenium (Se) [8]. Spinach is a dietary source of Ca, Cu, Fe, K, Mg, Mn, P, Zn, folate, vitamins and dietary fiber [9]. Therefore, breeding new spinach varieties with high nutritional components including the mineral elements is one of the main goals in spinach breeding programs worldwide.

Molecular plant breeding has been the foundation for twenty-first-century crop improvement [10]. Marker-assisted selection (MAS) has been successfully used to incorporate specific genes/alleles in plant breeding [11–13]. Single nucleotide polymorphism (SNP) with its high abundance, cost efficiency, and high-throughput scoring, has become a powerful tool in genome mapping, association studies, diversity analysis, and tagging of important genes in plant genomics [14–17]. Therefore, identification of SNP markers for mineral elements will be useful in spinach MAS breeding programs.

Genotyping by sequencing (GBS) is one of the next-generation sequencing platforms that utilizes a simple highly-multiplexed system for constructing reduced representation libraries which reduces sample handling, requires fewer PCR and purification steps, no size fractionation and uses inexpensive barcoding [18, 19]. As a cost-effective tool for MAS, GBS has been used to facilitate genome-wide association studies (GWAS), genetic linkage analysis, molecular marker discovery, and studies of genomic diversity or selection [18, 20, 21]. As the GBS method has no requirement for a priori knowledge of the species genomes, it has been shown to be robust across a range of species and SNP discovery and genotyping are completed together [22, 23]. The spinach genome assembly (PacBio Assembly) (980 Mbp) has been reported on January 14, 2014 [24, 25], but it has not been publically available yet. The spinach genome Spinach-1.0.1 is available to the public at http://www.ncbi.nlm.nih.gov/Traces/wgs/?val=AYZV02 and also at “The *Beta vulgaris* Resource” website with the page at http://bvseq.molgen.mpg.de/Genome/Download/Spinach/, representing approximately half of the spinach genome [26, 27]. We used the AYZV02 as the reference of spinach genome sequences for short reads assembly and SNP discovery in each spinach sample in this study.

To date, several association studies for different phenotypic traits of spinach have been reported, such as oxalate concentration [28], leafminer (*Liriomyza* spp.) resistance [29], Verticillium wilt resistance [30], Stemphylium leaf spot resistance [31], and leaf traits [32]. However, no genetic studies have been conducted to evaluate the genetic diversity of mineral elements and no research has been reported regarding mineral elements using association mapping in spinach natural populations to date. Therefore, the objectives of this study were to perform genetic diversity analysis and GWAS for spinach mineral elements in the USDA spinach germplasm collection, and to identify SNP markers associated with the 14 mineral elements: B, Ca, Co, Cu, Fe, K, Mg, Mn, Mo, Na, Ni, P, S, and Zn. The results will provide information on how to use spinach germplasm accessions with high mineral concentrations and new molecular markers for spinach breeding programs.

## Results

### Phenotypic variation in the mineral concentrations in spinach USDA-GRIN germplasm

The 14 mineral elements in spinach were analyzed for their mean (average) concentration, range, standard deviation (stdev), and coefficient of variation (CV) for each mineral element (Table 1). Potassium had the highest concentration with greater than 79,500 ppm; Mg the second with 8697 ppm; P, Ca, and S were greater than 4200 ppm; Na and Zn greater than 100 ppm; Mn, Fe, and B greater than 10 ppm; and Cu, Mo, Ni, and Co were less than 7 ppm, with Ni and Co with less than 1 ppm.

**Table 1:** The average, range, standard deviation, and coefficient of variation of the 14 mineral compounds in spinach

Mineral compound	No. sample	Average (ppm)	Minimum (ppm)	Maximum (ppm)	Range (ppm)	Standard deviation	Stdev error	CV(%)
K	275	79,576.06	47,170.00	108,935.36	61,765.37	11,903.06	43.28	14.96
Mg	292	8697.34	4921.23	14,293.27	9372.04	1443.39	4.94	16.60
P	292	6748.91	4004.33	10,210.77	6206.44	952.55	3.26	14.11
Ca	292	6400.55	3043.91	11,544.80	8500.89	1511.40	5.18	23.61
S	292	4281.57	1899.60	7462.84	5563.23	782.93	2.68	18.29
Na	292	615.76	186.94	1484.01	1297.07	214.52	0.73	34.84
Zn	292	102.50	31.43	386.82	355.38	78.97	0.27	77.04
Mn	292	83.20	29.78	415.76	385.97	79.00	0.27	94.95
Fe	291	74.34	50.43	138.58	88.15	13.57	0.05	18.25
B	269	30.47	17.07	52.36	35.29	5.93	0.02	19.47
Cu	292	6.86	2.22	10.97	8.75	1.25	0.00	18.16
Mo	292	2.36	0.20	8.62	8.42	1.29	0.00	54.96
Ni	288	0.72	0.23	4.23	4.00	0.48	0.00	66.29
Co	238	0.15	0.05	0.46	0.42	0.08	0.00	54.01

**Fig. 1:**
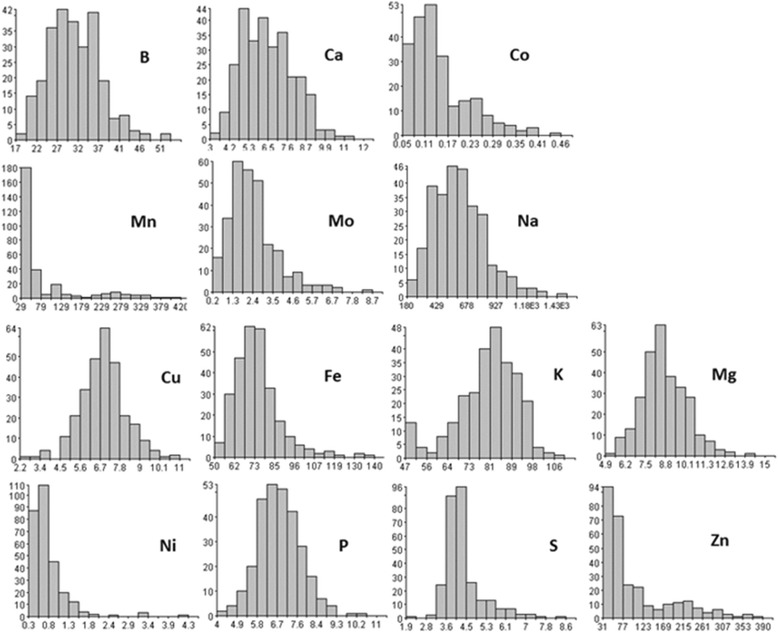
Distributions of the 14 mineral element concentrations in 292 USDA spinach germplasm accessions. The x-axis signifies the mineral concentrations and y-axis the number of spinach accessions, where the B, Co, Cu, Fe, Mn, Mo, Na, Ni, and Zn used μg/g dry weight (DW) and the Ca, K, Mg, P, and S used mg/g DW

All elements showed broad concentration ranges across the germplasm accessions. K, P, Fe, Mg, B, Ca, S, and Cu had about two to five times difference (Maximum/Minimum); Na, Co, Zn, and Mn showed approximately eight to fourteen times difference; while Ni (18 times) and Mo (42 times) exhibited the largest differences (Table 1). The standard deviation and stdev error showed similar trends with their averages. K had the highest average, and it also had the highest standard deviation and stdev error. The coefficient of variation (CV), also known as relative standard deviation (RSD), is a standardized measure of dispersion of a probability distribution or frequency distribution, where CV = mean/Stdev × 100. All 14 mineral elements had a large CV value of greater than 14%. Among them, Co, Mn, Mo, Ni, and Zn had greater than 50% CV value, indicated that the dispersion in the variable was greater (Table 1), showing there were large variations in these mineral elements across the spinach germplasm accessions. Analyzed from the distributions of mineral concentration values (Fig. 1), Co, Mn, Ni, and Zn showed a skewed distribution toward the lower range, and others had near-normal distributions.

**Fig. 2:**
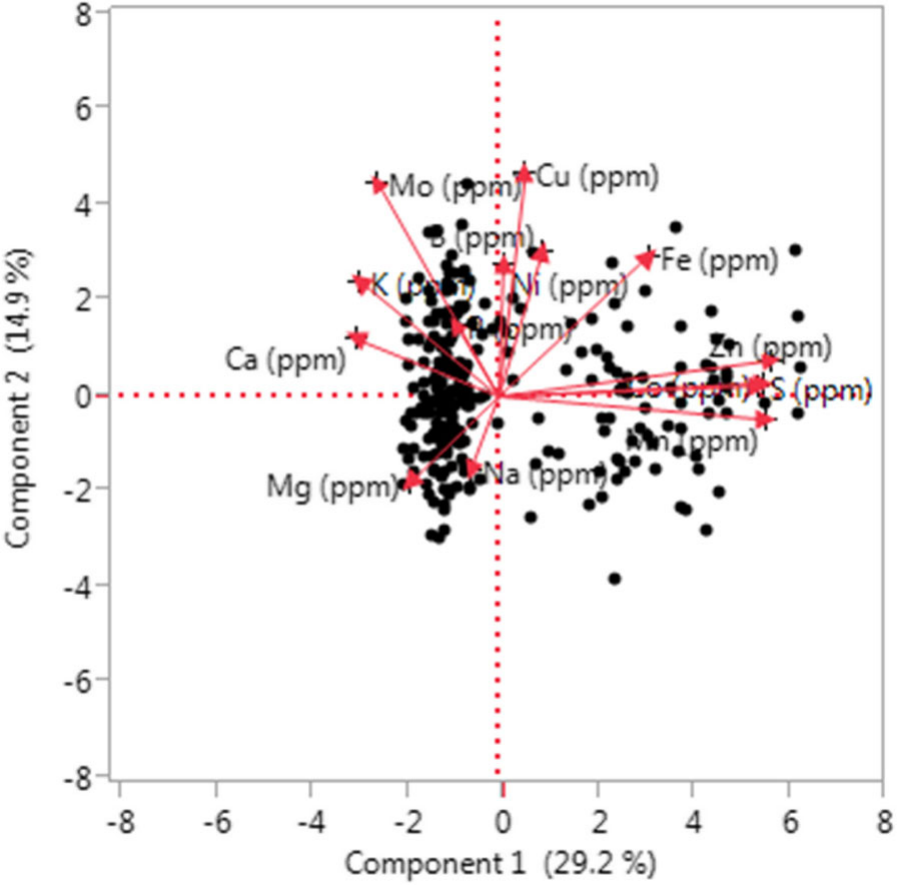
The two dimension plot of Biplot for 14 mineral element concentrations in 292 spinach germplasm accessions

The correlation among the 14 mineral elements was analyzed by JMP Genomics 7. The correlation coefficients among Co, Mn, S, and Zn were greater than 0.7, indicating strong associations (correlations) among the four mineral elements. In addition, the pairwise correlation coefficients of Fe and S, Fe and Co, Fe and Zn, Cu and Mo, K and P were greater than 0.4, indicating significant associations between each pair (Table 2). The two dimension plot of Biplot can be used to visualize the analysis of two–way data. As shown in Fig. 2, the lines that extend from the center and connect to each mineral element trait are considered as index vectors. The angles of the index vectors indicate that correlations existed between the indices (each mineral element trait). The cosine of these angles indicates the genetic correlation. An angle that is less than 90° indicates a positive correlation, and an angle that is greater than 90° indicates a negative correlation. If the angle is close to 0° or 180°, the two indices were highly correlated. The smaller angle between Co, Mn, S, and Zn, than other mineral elements, indicates their higher correlation than the other elements (Fig. 2).

**Table 2:** Correlation coefficient among fourteen mineral components in spinach

Correlation	B	Ca	Co	Cu	Fe	K	Mg	Mn	Mo	Na	Ni	P	S	Zn
B	1	0.09	0.12	0.22	0.11	0.09	−0.15	0.07	0.23	0.03	0.07	−0.01	0.15	0.23
Ca	0.09	1	−0.36	0.25	−0.07	0.01	0.37	−0.35	0.34	0.24	0.10	−0.27	−0.32	−0.37
Co	0.12	−0.36	1	0.14	0.44	−0.35	−0.21	0.72	−0.31	−0.04	0.02	−0.14	0.70	0.74
Cu	0.22	0.25	0.14	1	0.30	0.15	−0.16	0.02	0.44	−0.06	0.12	0.03	0.17	0.13
Fe	0.11	−0.07	0.44	0.30	1	−0.04	0.01	0.36	−0.02	−0.03	0.40	0.19	0.44	0.41
K	0.09	0.01	−0.35	0.15	−0.04	1	0.10	−0.36	0.31	−0.01	0.06	0.43	−0.32	−0.35
Mg	−0.15	0.37	−0.21	−0.16	0.01	0.10	1	−0.12	−0.22	0.35	−0.03	0.25	−0.12	−0.30
Mn	0.07	−0.35	0.72	0.02	0.36	−0.36	−0.12	1	−0.35	−0.02	−0.03	−0.09	0.76	0.81
Mo	0.23	0.34	−0.31	0.44	−0.02	0.31	−0.22	−0.35	1	−0.19	0.13	0.11	−0.34	−0.22
Na	0.03	0.24	−0.04	−0.06	−0.03	−0.01	0.35	−0.02	−0.19	1	0.00	−0.06	0.05	−0.16
Ni	0.07	0.10	0.02	0.12	0.40	0.06	−0.03	−0.03	0.13	0.00	1	0.08	−0.03	0.00
P	−0.01	−0.27	−0.14	0.03	0.19	0.43	0.25	−0.09	0.11	−0.06	0.08	1	−0.07	−0.11
S	0.15	−0.32	0.70	0.17	0.44	−0.32	−0.12	0.76	−0.34	0.05	−0.03	−0.07	1	0.72
Zn	0.23	−0.37	0.74	0.13	0.41	−0.35	−0.30	0.81	−0.22	−0.16	0.00	−0.11	0.72	1

### Genetic diversity analysis of spinach germplasm

**Fig. 3:**
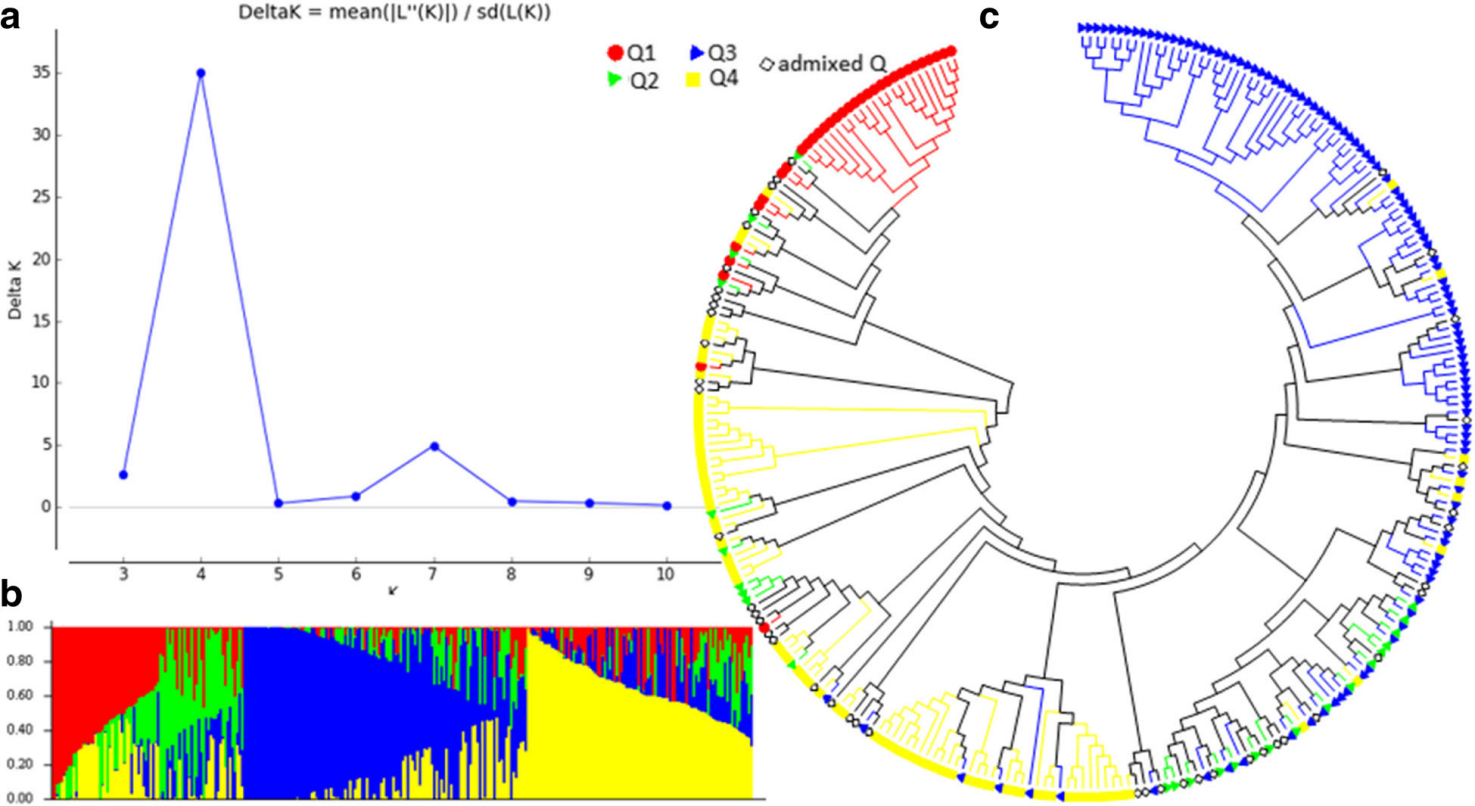
Model-based populations in association panels consisting of 292 USDA GRIN spinach germplasm accessions: **a** Delta K values for different numbers of populations (K) assumed in the analysis completed with the STRUCTURE software. **b** Classification of 292 spinach accessions into four populations using STRUCTURE Version 2.3.4, where the numbers on the y-axis show the subgroup membership, and the x-axis shows the different accession. The distribution of accessions into different populations is indicated by the color coding and shape (Cluster 1, Q1, is red round shape; Cluster 2, Q2, is the green triangle; Cluster 3, Q3, is the blue triangle; Cluster 4, and Q4, is yellow diamond). **c** Maximum Likelihood (ML) tree of the 292 spinach accessions drawn by MEGA 6. The color code for each population is consistent with (**b**) and (**c**), and the empty black square represents accessions aligned with the admixture cluster or population in 292 USDA GRIN spinach germplasm accessions

The population structure of the 292 spinach accessions was initially inferred using STRUCTURE 2.3.4 [33] and the peak of delta K was observed at K = 4, indicating the presence of the four main populations (clusters, Q1-Q4) in the 292 spinach accessions (Fig. 3a). The classification of accessions into populations based on the model-based structure was shown in Fig. 3 and Additional file 1: Table S1. In total, 247 accessions (84.6%) were assigned to one of the four populations (Q1, Q2, Q3, and Q4). Population 1, 2, 3, and 4 (Q1, Q2, Q3, and Q4) consisted of 33 (11.3%), 26 (8.9%), 109 (37.3%), and 79 (27.0%) accessions, respectively. The remaining 45 accessions (15.4%) were categorized as having admixed ancestry, including two, three, and four population admixed among Q1, Q2, Q3, and Q4 (Additional file 1: Table S1).

The genetic diversity was analyzed using the Maximum Likelihood (ML) method by MEGA 6 [34]. Several phylogenetic trees were drawn based on interpretation of results. We defined Q1, Q2, Q3, and Q4 as the clusters and used the same colors as the population structure Q1 (red), Q2 (green), and Q3 (blue), and Q4 (yellow) from the STRUCTURE 2.3.4 (Fig. 3b) to draw the subtrees of the phylogenetic trees in MEGA 6. Two phylogenetic trees were included: (1) without taxon names assigned in order to compare the populations from STRUCTURE (Fig. 3c), and (2) the ring phylogenetic tree (Additional file 2: Figure S1). The phylogenetic trees from MEGA 6 (Fig. 3c and Additional file 2: Figure S1), were well consistent with the structure populations (Q1-Q4) from STRUCTURE 2.3.4 (Fig. 3a and b), indicating that there were four well-differentiated genetic populations and admixture in the spinach panel.

### Association mapping and SNP marker identification

Six methods were used for association analysis of the 14 mineral elements among the 292 spinach accessions using 2401 SNPs. The six methods included: (1) SMR_Qgene: single marker regression using the QGene 4.3.10 [35], (2) SMR_tassel: single marker regression without structure and without kinship using TASSEL 5, (3) GLM_tassel: general linear model using TASSEL 5, (4) MLM_tassel: mixed linear model methods using TASSEL 5, (5) cMLM_gapit: compressed mixed linear model methods using GAPIT, and (6) EcMLM_gapit: enriched compressed mixed linear model methods using GAPIT. The selection standardization in this research is based on LOD values: SMR_QGene, SMR_tassel, and GLM_tassel > = 2.5, and one of MLM (either MLM_tassel, CMLM_gapit, or EcMLM_gapit) > = 2.5.

Based on the criteria above, a total of 45 SNPs were identified to be strongly associated with the 13 mineral elements except for K (Table 3). Among the 45 SNP markers, four were associated with B; one with Ca; seven with Co; two with Cu; six with Fe; four with Mg; five with Mn; one with Mo; five with Na; one with Ni; one with P; seven with S; and five with Zn. In addition, two SNP markers, AYZV02123399_305 and AYZV02147304_372, were detected to be associated with K having a LOD value > = 2.0 at SMR_QGene and SMR_tassel, LOD > = 2.5 at GLM_tassel, and one of MLM models (either MLM_tassel, CMLM_gapit, or EcMLM_gapit). Of these identified markers, four SNPs showed pleiotropic effects: AYZV02017731_35 was associated with both Co and S; AYZV02057049_393 with both Fe and S; AYZV02073631_255 with both Co and S; and AYZV02225779_181 with both Fe and S, indicating each of the four markers can be used to select two mineral components in MAS spinach breeding. Three SNP markers, AYZV02017731_40, AYZV02094133_57, and AYZV02281036_185 were identified to be associated with concentrations of four mineral components, Co, Mn, S, and Zn based on LOD values: SMR_Qgene (Table 4). T-test result validated the three SNP markers were significantly associated with the four mineral elements (Table 5), suggesting that it may be possible to select high contents of the four elements, Co, Mn, S, and Zn at the same time through MAS using these markers in breeding.

**Table 3:** The information of the significant SNP markers associated with 14 mineral compounds among the 292 spinach accessions using six statistical models, SMR_Qgene, SMR_tassel, GLM_tassel, MLM_tassel, CMLM_gapit, and EcMLM_gapit

Trait		LOD value (−LOG(p))						R-squre (%)				MAF
		Qgene	Tassel			GAPIT		Qgene	Tassel			
	Marker	SMR	SMR	GLM	MLM	cMLM	EcMLM	SMR	SMR	GLM	MLM	
B	AYZV02030222_447	5.15	4.59	3.75	2.94	3.23	3.18	8.40	8.12	6.36	5.40	14.37
B	AYZV02101102_298	2.67	2.59	3.08	3.32	3.65	3.61	4.50	4.47	5.06	5.92	11.75
B	AYZV02164196_389	3.61	4.02	3.15	2.69	3.45	3.36	6.00	7.51	5.75	5.23	20.34
B	AYZV02164196_391	3.31	3.48	2.66	2.13	2.66	2.58	5.50	6.54	4.88	4.10	20.34
Ca	AYZV02244039_336	2.53	3.13	3.04	2.70	0.17	0.40	3.90	5.79	5.65	5.36	27.32
Co	AYZV02017731_35	5.64	5.75	4.16	3.77	2.99	2.38	10.30	9.80	6.26	6.15	9.92
Co	AYZV02073631_257	3.86	2.70	2.62	1.68	2.73	3.52	7.20	5.35	4.76	3.35	6.75
Co	AYZV02165009_136	3.28	3.41	3.03	2.67	2.90	2.14	6.20	5.37	4.31	4.03	9.07
Co	AYZV02217527_245	3.74	4.15	3.17	2.15	4.34	4.53	7.00	6.58	4.44	3.15	2.74
Co	AYZV02217549_245	3.74	3.65	2.53	1.83	2.59	2.69	7.00	6.96	4.43	3.45	3.38
Co	AYZV02220844_249	3.04	3.60	3.49	3.13	3.53	2.06	5.70	6.99	6.18	6.05	10.97
Co	AYZV02221073_190	3.41	4.18	3.19	2.96	2.28	2.10	6.40	7.15	4.81	4.80	23.63
Cu	AYZV02147304_383	3.01	3.27	3.62	3.01	3.27	3.30	4.60	5.22	5.62	4.98	14.09
Cu	AYZV02277499_79	2.75	3.45	4.67	3.66	3.21	3.33	4.20	5.76	7.49	6.43	43.30
Fe	AYZV02057049_393	4.68	4.61	4.90	4.41	0.10	0.12	7.10	7.33	7.54	7.35	11.90
Fe	AYZV02201149_106	2.85	4.08	5.48	4.74	0.44	0.10	4.40	7.43	9.36	9.37	23.45
Fe	AYZV02201149_51	2.65	3.01	3.59	3.21	0.45	0.05	4.10	5.58	6.27	6.27	24.14
Fe	AYZV02207926_4318	3.88	4.45	3.35	3.05	1.20	1.12	6.00	7.47	5.37	5.52	18.79
Fe	AYZV02225779_181	5.87	5.67	6.38	5.74	3.39	3.38	8.90	8.97	9.71	9.56	5.00
Fe	AYZV02296000_81	3.46	4.53	4.28	3.89	0.92	0.21	5.30	7.65	7.03	6.76	23.45
Mg	AYZV02113550_76	3.09	3.66	3.33	2.64	3.12	2.00	4.70	5.74	4.95	4.13	41.41
Mg	AYZV02144992_13	5.38	3.98	3.17	0.96	1.80	2.78	8.10	6.93	5.45	1.84	18.56
Mg	AYZV02176946_248	3.98	5.05	4.50	3.48	1.29	1.52	6.10	7.84	6.89	5.93	12.71
Mg	AYZV02297745_849	4.21	5.92	6.03	3.32	1.02	3.75	6.40	8.07	8.06	4.62	39.52
Mn	AYZV02026295_260	4.79	4.68	3.79	2.76	1.22	1.11	7.30	7.26	5.87	4.52	5.33
Mn	AYZV02136507_242	7.08	9.20	8.32	6.68	0.91	0.52	10.60	15.30	13.77	12.90	43.81
Mn	AYZV02136507_248	5.18	6.49	5.66	4.81	0.32	0.02	7.90	11.26	9.76	9.20	41.41
Mn	AYZV02245160_278	3.28	3.36	2.82	3.03	0.08	0.56	5.00	5.72	4.77	5.43	17.18
Mn	AYZV02281036_185	3.12	3.45	3.01	2.88	2.97	1.95	4.80	5.69	4.95	5.14	8.59
Mo	AYZV02092600_720	3.15	3.77	4.04	3.88	3.45	3.76	4.80	4.82	5.05	5.10	4.81
Na	AYZV02103789_734	3.52	3.65	2.87	2.50	2.10	2.06	5.40	4.93	3.61	3.17	13.57
Na	AYZV02156839_631	3.08	3.11	2.86	2.68	0.02	0.07	4.70	5.30	4.77	4.93	23.54
Na	AYZV02178194_42	3.38	4.11	2.70	2.08	2.57	1.84	5.20	6.23	3.70	2.84	28.87
Na	AYZV02209560_3	3.77	4.59	3.42	2.84	3.16	3.31	5.80	6.29	4.40	3.72	13.40
Na	AYZV02225745_194	3.05	3.21	2.59	2.24	1.93	2.51	4.70	4.48	3.42	2.91	18.56
Ni	AYZV02051025_93	3.40	6.42	5.77	4.87	0.30	0.31	5.30	10.33	9.23	8.71	28.92
P	AYZV02105368_125	2.88	3.40	3.54	2.82	2.91	2.80	4.40	5.82	6.05	5.09	11.00
S	AYZV02017731_35	3.84	4.33	3.39	2.84	2.08	1.38	5.90	5.92	4.39	3.71	10.14
S	AYZV02057049_393	3.78	3.88	4.21	3.64	0.29	0.07	5.80	6.18	6.55	6.19	11.86
S	AYZV02073631_255	3.58	2.69	2.83	2.52	3.04	2.82	5.50	4.40	4.53	4.28	6.70
S	AYZV02073631_257	3.65	2.79	2.88	2.57	3.34	3.05	5.60	4.54	4.59	4.35	6.36
S	AYZV02094133_57	5.36	6.48	6.15	4.96	3.06	2.46	8.10	9.70	8.95	7.87	16.32
S	AYZV02225779_181	4.28	4.12	4.45	3.87	0.01	0.03	6.50	6.57	6.92	6.37	4.98
S	AYZV02248538_132	2.75	3.60	3.49	3.74	1.22	1.08	4.20	5.93	5.61	6.35	9.28
Zn	AYZV02066684_19177	3.71	3.24	3.19	2.72	0.66	0.57	5.70	5.46	4.97	5.44	14.95
Zn	AYZV02151846_237	3.06	5.43	3.09	2.17	2.53	1.71	4.70	8.58	4.29	3.49	36.08
Zn	AYZV02201848_2665	5.11	5.16	4.30	3.96	2.69	2.72	7.70	8.14	6.34	6.59	11.34
Zn	AYZV02212287_92	2.54	3.92	4.91	3.15	1.66	1.46	3.90	6.57	7.41	5.26	17.01
Zn	AYZV02212288_92	3.30	4.13	5.27	3.69	2.53	2.32	5.10	7.19	8.27	6.19	19.59
K	AYZV02123399_305	2.07	2.25	2.77	2.61	2.53	2.80	3.40	4.05	4.94	4.84	25.00
K	AYZV02147304_372	2.33	2.21	2.48	2.53	2.09	1.69	3.80	3.90	4.36	4.47	18.43

**Table 4:** The information of three significant SNP markers associated with four mineral compounds among the 292 spinach accessions using six statistical models, SMR_Qgene, SMR_tassel, GLM_tassel, MLM_tassel, CMLM_gapit, and EcMLM_gapit. Co, Mn, S, and Zn based on T-tesing at *P* = 0.05

Trait		LOD value (−LOG(p))						R-squre (%)				MAF
		Qgene	Tassel			GAPIT		Qgene	Tassel			
	Marker	SMR	SMR	GLM	MLM	cMLM	EcMLM	SMR	SMR	GLM	MLM	
Co	AYZV02017730_40	3.74	3.83	2.35	1.98	2.26	1.55	7.00	6.12	3.16	2.78	7.17
Mn	AYZV02017730_40	2.06	2.65	2.10	1.15	0.99	0.66	3.20	3.28	2.46	1.17	6.53
S	AYZV02017730_40	3.25	3.95	3.09	2.42	2.22	1.47	5.00	5.17	3.83	3.01	6.53
Zn	AYZV02017730_40	3.35	3.93	2.63	2.04	1.80	1.19	5.10	5.15	2.98	2.51	6.53
Co	AYZV02094133_57	2.07	2.53	1.46	1.27	1.08	0.65	3.90	4.18	1.93	1.78	16.67
Mn	AYZV02094133_57	4.04	4.50	3.49	2.16	0.33	0.63	6.20	6.56	4.88	2.98	16.32
S	AYZV02094133_57	5.36	6.48	6.15	4.96	3.06	2.46	8.10	9.70	8.95	7.87	16.32
Zn	AYZV02094133_57	1.62	2.30	1.99	1.13	0.49	0.17	2.50	3.04	2.35	1.28	16.32
Co	AYZV02281036_185	2.29	1.54	1.12	1.06	2.37	1.41	4.30	3.17	2.12	2.23	9.07
Mn	AYZV02281036_185	3.12	3.45	3.01	2.88	2.97	1.95	4.80	5.69	4.95	5.14	8.59
S	AYZV02281036_185	2.35	2.00	1.70	1.75	1.71	1.35	3.60	3.34	2.78	3.03	8.59
Zn	AYZV02281036_185	2.86	2.85	2.60	2.47	3.19	2.76	4.40	4.73	3.97	4.22	8.59

**Table 5:** Three SNP markers significantly associated with two to four of the four mineral compound, Co, Mn, S, and Zn based on T-tesing at P = 0.05

SNP	Co			Mn			S			Zn		
	Allele	Significant	LSM	Allele	Significant	LSM	Allele	Significant	LSM	Allele	Significant	LSM
AYZV02017730_40				GG	A	320.375	GG	A	6539.647	GG	A	287.886
AYZV02017730_40				CC	B	235.260	CC	B	5550.364	CC	B	189.567
AYZV02094133_57				CC	A	302.734	CC	A	6461.113			
AYZV02094133_57				AA	B	256.411	AA	B	5835.309			
AYZV02281036_185	AA	A	0.428	AA	A	283.587	AA	A	6397.044	AA	A	306.576
AYZV02281036_185	CC	B	0.384	CC	B	219.221	CC	B	5847.326	CC	B	240.304
AYZV02281036_185	AC	AB	0.366	AC	AB	361.036	AC	AB	6037.149	AC	AB	215.736

R-square (Rsq) of the detected markers using the six different methods were listed in Table 3 with a large range from 1.84% of the AYZV02144992_13 marker, associated with Mg in MLM model, to 15.3% of the AYZV02136507_242, associated with Mn in SMR from Tassel. According to the LOD value, the larger Rsq value of a marker, the stronger association is the marker which makes more contribution to the trait. In all detected SNP markers, AYZV02136507_242 had the greatest Rsq value with 10.6% Rsq in SMR model from QGene, 15.3% Rsq in SMR, 13.8% Rsq in GLM, and 12.9% Rsq in GLM from Tassel for Mn element, indicating that the AYZV02136507_242 is strongly associated with Mn from this study.

### Evaluation and genetic diversity analysis of the top three spinach germplasm accessions for each mineral

We identified the top 3 accessions with the highest concentrations in each of the 14 mineral elements. First, we ordered the 292 spinach accessions based on their mineral concentrations by each individually from the highest to lowest values and gave the order number from 1 to 292 plus the mineral name for each mineral element. For B, as an example, we ordered the B concentration from the highest to lowest and gave each accession with an order ID from B1 to B292 such as NSL6557 had the highest B value with 52.36 ppm and was given B1, and NSL6083 the lowest B value with 17.07 ppm given B292 (Additional file 1: Table S1). By combining the 14 mineral elements, each accession was given a mineral concentration order ID including the 14 mineral names and their order numbers. For example, the accession NSL6557 was given the designation B1Ca63Co125Cu41Fe133K284Mg34Mn67Mo36Na161Ni41P19S72Zn100, which means a B rank of 1, Ca rank of 63, Co rank of 125, Cu rank of 41, Fe rank of 133, etc. for this accession (Additional file 1: Table S2). After ranking all 14 mineral elements, 31 spinach accessions had been chosen because they had at least one mineral element (out of 14) ranked in the top three among the 292 spinach accessions (Additional file 1: Table S2), indicating that the 31 accessions were good mineral element resources for spinach breeding to improve mineral concentrations. Eight out of these 31 spinach accessions had more than two mineral elements ranked in the top 3 highest (PI604777, PI604786, PI175595, PI604782, PI360895, PI339547, PI176372, and PI169671). Three out of the eight accessions had three mineral elements ranked in the top 3 highest. PI604777 was ranked as No. 1 in S, No. 2 in Fe, and No. 3 in Co; PI604786 as No. 2 in Zn, No. 2 in Mn, and No. 3 in Cu; and PI175595 was No. 1 in Ni, No. 3 in P, and No. 3 in K (Additional file 1: Table S2).

**Fig. 4:**
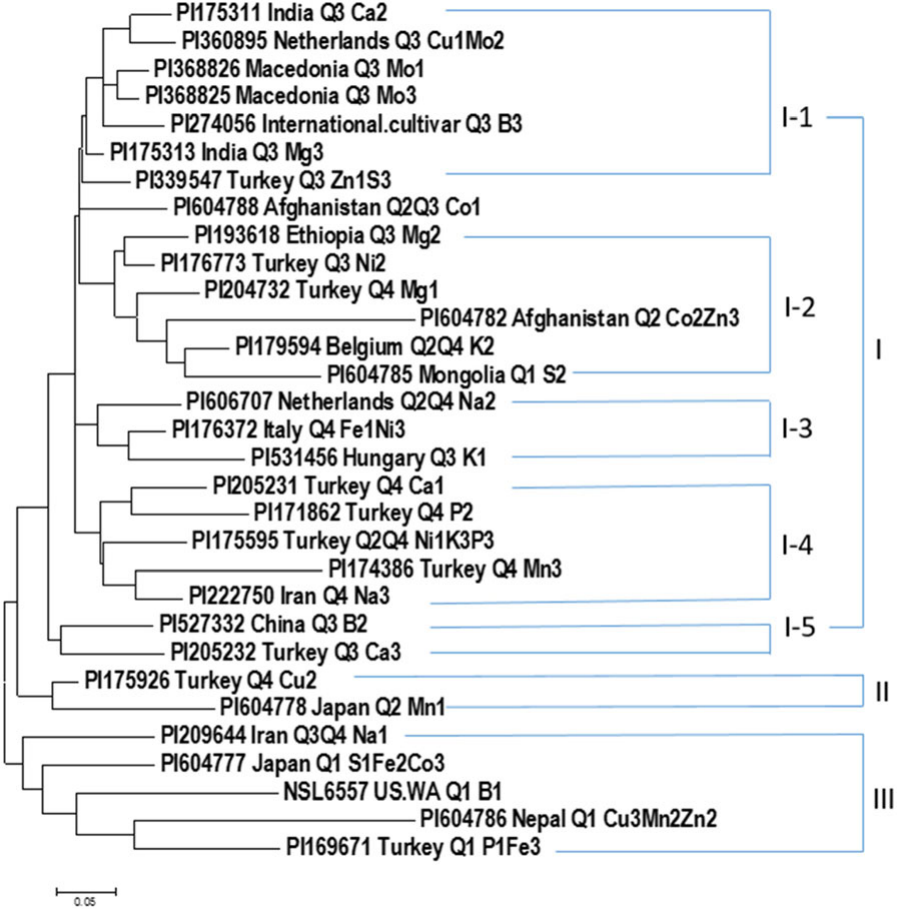
The ring phylogenetic tree created by the Maximum Likelihood (ML) method from MEGA 6 in 31 spinach germplasm accessions that had at least one mineral element ranked in the top three highest concentration among the 292 spinach accessions for 14 mineral elements

The 31 spinach accessions were collected from 10 countries plus one unknown location: ten from Turkey, two from Afghanistan, India, Iran, Japan, Macedonia, and Netherlands, respectively, and one from Mongolia, Belgium, China, Ethiopia, Hungary, Italy, Nepal, and the US, respectively (Additional file 1: Table S2), The genetic diversity analysis was performed for the 31 spinach accessions and the phylogenetic tree was drawn using MEGA 6 (Fig. 4). Based on genetic distances among the 31 genotypes, there were three clusters: Cluster I, consisted of 24 accessions; Cluster II had only two accessions; and Cluster III, included five accessions. The cluster I can be further divided into five sub-clusters (groups): I-1 with seven accessions, I-2, six accessions, I-3, three accessions, I-4, five accessions, and I-5, only two accessions. In addition, PI604788 is an outlier (Fig. 4). The genetic diversity and phylogenetic analysis will provide the information for breeders to choose these spinach accessions as parents in breeding programs.

## Discussion

### Application of marker-assisted selection in the genetic improvement of spinach breeding

In this study, we conducted a comprehensive GWAS to identify genetic loci with SNP markers associated with the 14 mineral elements (B, Ca, Co, Cu, Fe, K, Mg, Mn, Mo, Na, Ni, P, S, and Zn) in 292 spinach accessions of the USDA collection. A total of 45 SNP markers were identified to be associated with the 13 mineral elements except K, based on six different association mapping models. Similar mineral element association mapping research has been reported in other crops, such as pea [36] and rice [37–40] using RIL or diversity populations. Ma et al. (2017) [36] conducted a comprehensive QTL mapping study and identified genetic loci associated with mineral element concentrations (B, Ca, Fe, K, Mg, Mn, Mo, P, S and Zn) in pea seeds using a RIL population.

Co-localization of SNPs for different element concentrations has been discovered in this research. AYZV02017731_35 and AYZV02073631_257 were related to Co and S, AYZV02057049_393 was related to both Fe and S, and AYZV02225779_181 was related to Fe and S (Table 3). Co-localization of QTLs for different element concentrations in seeds has previously been reported in rice [37, 38, 41]. Genetically, the phenomenon of co-localization may be caused by pleiotropy of a single gene product being involved in the transport and/or physiological processing of multiple elements. Another possibility is the presence of clustered genes that are tightly associated together and responsible for the accumulation of different elements [42].

Accordingly, the high positive correlations among Co, S, Mn, and Zn ranged from 0.70 to 0.81 in this study (Table 2). The positive correlations suggest that high Co, S, Mn and Zn concentrations could be possible in individual spinach accessions. For the four mineral elements (Co, S, Mn and Zn), eleven highly significantly associated SNP markers were identified in this study (Table 3).

These markers related to mineral elements have low LOD and small R-squared values because the mineral elements are controlled by multiple genes with minor effects. How to apply these SNP markers in spinach germplasm evaluation and breeding is a challenge for spinach breeders. Next step of research is going to validate these SNP markers using KASP SNP genotyping: 1) To validate the stability of these SNP markers across multiple locations and years; 2) To conduct an additional association mapping study on another natural population panel in order to identify potential overlaps with what we are reporting; 3) To develop bi-parental populations to validate and identify SNP markers linked to mineral elements and try to find SNP markers with major effect; 4) To use genomic selection approach to select the minor effect alleles to improve element compositions in spinach cultivars.

This comprehensive spinach mineral nutrient study provides a foundation of SNP markers to improve mineral content in spinach cultivar development. The future release of a spinach reference genome sequence will enable a complete analysis of these trait loci.

### Mineral elements related to nutrition and human healthy food in spinach

The mineral elements are essential and indispensable for growth and health, having a direct or indirect effect on the metabolism and physiological processes of humans and plants as well. Deficiencies or insufficient intake of minerals may lead to several dysfunctions and diseases in humans [43]. There is a growing interest in the mineral and phytochemical composition of foods and diets, and especially in leafy vegetables. Recent studies with Mexican, Central American, and African green leafy vegetables, including *Cnidoscolus aconitifolius*, *Crotalaria longirostrata*, *Solanum scabrum*, *Gynandropsis gyandra*, and several leafy *Amaranthus* species, have highlighted the contributions that these vegetables can provide to one’s daily intake of essential nutrients and health-beneficial compounds [44, 45].

Mineral elements in spinach have been reported by other studies employing a large number of techniques [46–51]. On a moisture-free basis, the highest levels of K, Ca, Na, P, Mg, S, Zn, Cu, Co, and B were found in spinach compared to parsley, dill, and mint [52]. In this research, 14 mineral elements were detected in 292 spinach accessions, including macro-elements (K, Mg, P, Ca, and S) and micro-elements (Na, Zn, Mn, B, Fe, Cu, Mo, Co, and Ni). These findings provide a new range of spinach mineral values for dieticians and nutrition scientists to consider when calculating nutrient intakes of spinach-containing diets, or when developing healthful menu recommendations.

Na, K, Mg, and Ca are the four main and essential electrolytes for humans, Na is responsible for controlling the total amount of water in the body. It is also important for regulating blood volume and maintaining muscle and nerve function. Mg is the most abundant intracellular divalent cation. It is an essential cofactor for a multitude of enzymatic reactions that are important for the generation of energy from ATP and for physiologic processes, including neuromuscular function and maintenance of cardiovascular tone [53]. Ca is the major component of bone and assists in tooth development [54]. K is an important component of cell and body fluids that helps to control heart rate and blood pressure (http://www.nutrition-and-you.com/spinach.html). Among the 292 spinach accessions from this research, the highest levels of Na, K, Mg, and Ca were found to have 1484.01 ppm, 108,935.36 ppm, 14,293.27 ppm, and 10,210.76 ppm in PI209644 from Iraq, PI531456 from Hungary, PI204732 from Turkey, and PI205231 from Turkey, respectively (Additional file 1: Table S1). In addition, the top three spinach germplasm accessions in each mineral element were also listed and provide information on how to use these high mineral accessions in breeding programs. The PI accessions containing beneficial SNPs associated with mineral elements will be highly valuable for the spinach breeders to use for the development of cultivars with high mineral element concentrations through MAS and GS (genomic selection). The significant genetic variations among genotypes as evidenced by the 2 to 42 times difference in mineral concentration (Table 1) suggest that the genetic improvement of mineral traits is feasible in spinach. The co-localization of SNP markers and the positive correlations in concentrations for many mineral elements make it possible to pyramid high concentrations of multiple elements into a single cultivar in a spinach breeding program.

## Conclusions

A total of 14 mineral elements: boron (B), calcium (Ca), cobalt (Co), copper (Cu), iron (Fe), potassium (K), magnesium (Mg), manganese (Mn), molybdenum (Mo), sodium (Na), nickel (Ni), phosphorus (P), sulfur (S), and zinc (Zn) were evaluated in 292 spinach accessions originally collected from 29 countries. The 45 SNP markers strongly associated with the concentrations of the 13 mineral elements: B, Ca, Co, Cu, Fe, Mg, Mn, Mo, Na, Ni, P, S, and Zn using six statics methods, including single marker regression using Q-gene (SMR), single marker regression using Tassel (SMR), general linear model using Tassel (GLM), mixed linear model using Tassel (MLM), compressed mixed linear model using Gapit (cMLM), and enriched compressed mixed linear model using Gapit (EcMLM). Three SNP markers, AYZV02017731_40, AYZV02094133_57, and AYZV02281036_185 were identified to be associated with concentrations of four mineral components, Co, Mn, S, and Zn. The markers could be used in breeding programs to improve the nutritional quality of spinach through marker-assisted selection (MAS). Thirty-one spinach accessions with high concentrations of one to several mineral elements can be used as potential parents for spinach breeding programs*.*

## Methods

### Plant materials

A total of 292 accessions of spinach (*S. oleracea*) USDA-GRIN (US Department of Agriculture, Germplasm Resources Information Network) germplasm originally collected from 29 countries plus 18 unknown locations were used for genetic diversity and association analysis of mineral elements in this study (Additional file 1: Table S1). All seeds were kindly provided by David Brenner at USDA-ARS (Agricultural Research Service) and Iowa State University at Ames, IA, US.

### Leaf mineral concentration evaluation

Concentrations of 14 mineral components: B, Ca, Co, Cu, Fe, K, Mg, Mn, Mo, Na, Ni, P, S, and Zn were evaluated in 292 USDA spinach germplasm accessions (Additional file 1: Table S1). The phenotypic data of the nine among the 14 elements in spinach germplasm accessions, including Ca, Cu, Fe, Mg, Mn, Mo, Ni, P, and Zn, have been published partially in the USDA GRIN website at http://www.ars-grin.gov/cgi-bin/npgs/html/eval.pl?492376.

Accessions were grown in 1-l black plastic pots filled with a 2:1 (vol: vol) mixture of commercially available soil (Metro-Mix 360; Scotts-Sierra Horticultural Products Co., Marysville, Ohio, USA) and vermiculite (Strong-Lite Medium Vermiculite; Sun Gro Horticulture Co, Seneca, Illinois, USA). There were six plants of each accession in a pot, with pots randomly distributed within a growth chamber (model PGW36; Controlled Environments Ltd., Winnipeg, Manitoba, Canada). Plants were grown on a 12 h. photoperiod of 300 μmol m^−2^ s^−1^ photosynthetically active radiation (incandescent and fluorescent lamps) with a 20 ± 0.5 °C / 15 ± 0.5 °C day/night temperature regime. Relative humidity was maintained at 50% ± 5%. Pots were initially irrigated with deionized water, and after emergence, plants were subirrigated daily with a nutrient solution containing the concentrations of mineral salts: 1.2 mM KNO_3_, 0.8 mM Ca(NO_3_)_2_, 0.8 mM NH_4_NO_3_, 0.2 mM MgSO_4_, 0.3 mM KH_2_PO_4_, 25 μM CaCl_2_, 25 μM H_3_BO_3_, 2 μm MnSO_4_, 2 μM ZnSO_4_, 0.5 μM CuSO_4_, 0.5 μM H_2_MoO_4_, 0.1 μM NiSO_4_, and 10 μM Fe(Ш)-N, N′-ethylenebis[2-(2-hydroxyphenyl)-glycine] (Sprint 138; Becker-Underwood, Inc., Ames, Iowa, USA).

Plants were harvested at 4–5 weeks after planting when they had 5–6 fully expanded leaves. Harvested material included both mature and immature leaves (leaf blades and petioles) from the six plants of each accession. Soil contamination of the samples was minimized by cutting plants 0.5 cm above the soil surface. Leaves were dried in paper bags at 65–70 °C for a minimum of 48 h, and after cooling the pooled leaves from the 6 plants were ground to a uniform powder using a coffee grinder with stainless steel blades (model IDS 55; Mr. Coffee, Boca Raton, Florida, USA). Two 0.25 g (dry weight) subsamples of each accession were wet digested in borosilicate glass tubes using ultra-pure nitric and perchloric acids, as previously described [55]. Digestages were resuspended in ultra-pure nitric acid and analyzed for concentrations of B, Ca, Co, Cu, Fe, K, Mg, Mn, Mo, Na, Ni, P, S, and Zn using inductively coupled plasma optical emission spectrometry (CIROS ICP Model FCE12; Spectro, Kleve, Germany). Tomato leaf standards (SRM 1573A; National Institute of Standards and Technology, Gaithersburg, Maryland, USA) were digested and analyzed as quality control along with each run of 50 spinach samples to verify the reliability of the procedures and analytical measurements. Results are reported on a dry weight basis as the average of the two subsamples in ppm (parts per million; equivalent to micrograms per gram).

### DNA extraction, GBS, and SNP discovery

Genomic DNA was extracted from freeze-dried fresh leaves of spinach plants using the CTAB (hexadecyltrimethyl ammonium bromide) method [56]. DNA sequencing was done by next generation sequencing technologies using GBS [18, 20] and GBS was conducted by HiSeq 2000 in Beijing Genome Institute (BGI). SOAP family software (http://soap.genomics.org.cn/) was used for sequence assembly, mapping and SNP discovery of GBS data. The GBS data averaged 3.26 M short-read and 283.74 Mbp data-points for each spinach sample. The short reads of the GBS data were aligned to spinach genome reference AYZV02 (http://www.ncbi.nlm.nih.gov/Traces/wgs/?val=AYZV02) by using SOAPaligner/soap2 (http://soap.genomics.org.cn/), while SOAPsnp v. 1.05 was used for SNP calling [57, 58]. Approximately a half million SNPs were discovered from the original GBS data from BGI among the 292 spinach germplasm accessions. The spinach accessions and SNPs were filtered before conducting genetic diversity and association analyses. If an accession had greater than 35% missing SNP data, the accession was removed from the panel. The SNP data were filtered by setting the parameters of minor allele frequency (MLF) > 2%, missing data <20%, and heterozygous genotype <10%. After filtering, 2402 SNPs among 292 spinach accessions were used for genetic diversity and association analysis.

### Phenotypic data analysis

Phenotypic data of the 14 mineral elements in spinach were analyzed using Microsoft Excel 2016 for the average, range, standard deviation, and coefficient of variation (CV) and the distributions of the 14 mineral elements were drawn using QGene [35]. The correlation coefficients of the 14 mineral elements were calculated using JMP Genomics 7 software (SAS Institute, Cary, NC, USA). In JMP Genomics 7, the dendrogram construction was done using multivariate methods to do hierarchical clustering. After being clustered, the multivariate principal component analysis (PCA) was used to create biplot on covariance.

### Genetic diversity and population structure analysis

The model-based program STRUCTURE 2 [33] was used to infer population structure. In order to identify the number of populations (K) capturing the major population structure in the tested spinach association panel, the burn-in period was set at 50,000 with the Markov Chain Monte Carlo iterations and the run length was set at 10,000 in an admixture model and correlated allele frequencies independent for each run [59]. Ten runs were performed for each simulated value of K, ranging from 1 to 10. The delta K was calculated using the formula provided by Evanno et al. (2005) [60]. The optimal K was determined with Structure Harvester [61]. After the optimal K was determined, a Q-matrix was generated; this was used in Tassel 5 for association analysis of mineral elements. Each spinach accession was also assigned to a cluster (Q) based on a probability for that accession in a cluster, using a cut-off probability of 0.50. Based on the optimal K, a Bar plot with ‘Sort by Q’ was obtained to visualize the population structure of the spinach association panel.

Genetic diversity was also assessed and the phylogeny trees were drawn using MEGA 6 [34] based on the Maximum Likelihood tree method with the following parameters [17]. Test of Phylogeny: Bootstrap Method, No. of Bootstrap Replications: 500, Model/Method: General Time Reversible model, Rates among Sites: Gamma distributed with Invariant sites (G + I), Number of Discrete Gamma Categories: 5, Gaps/Missing Data Treatment: Use all sites, ML Heuristic Method: Subtree-Pruning-Regrafting-Extensive (SPR level 5), Initial Tree for ML: Make initial tree automatically (Neighbor Joining), and Branch Swap Filter: Moderate. During the drawing of the phylogeny trees, the population structure and the cluster information were imported to MEGA 6 for combined analysis of genetic diversity. For sub-tree of each Q (cluster), the shape of ‘Node/Subtree Marker’ and the ‘Branch Line’ was drawn with the same color as in the figure of the Bar plot of the population clusters from the STRUCTURE analysis.

### Association analysis

Association analysis was conducted with the single marker regression (SMR) without structure and kinship, the general linear model (GLM), and the mixed linear model (MLM) methods as described in TASSEL 5 [62] (http://www.maizegenetics.net/tassel) and the analysis was also performed with compressed mixed linear model (cMLM) [63] and enriched compressed mixed linear model (EcMLM) [64] implemented in the GAPIT R package [65]. The QGene 4.3.10 was also used to conduct SMR for all SNPs [35], although QGene was developed for QTL mapping, it can also be used in association analysis through SMR. The effect of SNP markers were also conducted by T-test using JMP Genomics and Microsoft Excel 2016.

## Additional files

Additional file 1: Table S1. Spinach PI accession number, name, origin/country, cluster assigned in this study, taxon name, and 14 mineral element concentrations in 292 germplasm accessions. **Table S2.** Spinach PI accession number, taxon name, origin country, cluster assigned in this study, mineral element, mineral ID ranked in top three, and 14 mineral element concentrations in 292 germplasm accessions. (XLSX 105 kb)

Additional file 2: Figure S1. The ring phylogenetic tree combining structure populations (Q1 to Q4) from STRUCTURE 2 and the Maximum Likelihood (ML) method from MEGA 6. The spinach accession number, the accession original country, and the structure population (cluster) were merged together into one taxon name as each spinach accession ID in the combined tree drawn by MEGA 6. The colored shape and branch of each cluster matched the structure population (red round shape for Q1, green triangle for Q2, blue triangle for Q3, yellow diamond for Q4, and the black square with the black branch for the admixture in 292 USDA GRIN spinach germplasm accessions. (XLSX 149 kb)

## Abbreviations

B: Boron
Ca: Calcium
CMLM: Compressed mixed linear model using Gapit
Co: Cobalt
Cu: Copper
EcMLM: Enriched compressed mixed linear model using Gapit
Fe: Iron
GBS: Genotyping by sequencing
GLM: General linear model
GWAS: genome-wide association study
K: Potassium
MAS: Marker-assisted selection
Mg: Magnesium
MLF: Minor allele frequency
MLM: Mixed linear model
Mn: Manganese
Mo: Molybdenum
Na: Sodium
Ni: Nickel
P: Phosphorus
S: Sulfur
SMR: Single marker regression
SNP: Single nucleotide polymorphism
Zn: Zinc
